# Assisting allied health in performance evaluation: a systematic review

**DOI:** 10.1186/s12913-014-0572-7

**Published:** 2014-11-14

**Authors:** Lucylynn Lizarondo, Karen Grimmer, Saravana Kumar

**Affiliations:** International Centre for Allied Health Evidence, School of Health Sciences, University of South Australia, Adelaide, South Australia

**Keywords:** Performance evaluation, Allied health, Quality in health care, Process, Outcomes, Health services

## Abstract

**Background:**

Performance evaluation raises several challenges to allied health practitioners and there is no agreed approach to measuring or monitoring allied health service performance. The aim of this review was to examine the literature on performance evaluation in healthcare to assist in the establishment of a framework that can guide the measurement and evaluation of allied health clinical service performance. This review determined the core elements of a performance evaluation system, tools for evaluating performance, and barriers to the implementation of performance evaluation.

**Methods:**

A systematic review of the literature was undertaken. Five electronic databases were used to search for relevant articles: MEDLINE, Embase, CINAHL, PsychInfo, and Academic Search Premier. Articles which focussed on any allied health performance evaluation or those which examined performance in health care in general were considered in the review. Content analysis was used to synthesise the findings from individual articles.

**Results:**

A total of 37 articles were included in the review. The literature suggests there are core elements involved in performance evaluation which include prioritising clinical areas for measurement, setting goals, selecting performance measures, identifying sources of feedback, undertaking performance measurement, and reporting the results to relevant stakeholders. The literature describes performance evaluation as multi-dimensional, requiring information or data from more than one perspective to provide a rich assessment of performance. A range of tools or instruments are available to capture various perspectives and gather a comprehensive picture of health care quality.

**Conclusions:**

Every allied health care delivery system has different performance needs and will therefore require different approaches. However, there are core processes that can be used as a framework to evaluate allied health performance. A careful examination of barriers to performance evaluation and subsequent tailoring of strategies to overcome these barriers should be undertaken to achieve the aims of performance evaluation. The findings of this review should inform the development of a standardised framework that can be used to measure and evaluate allied health performance. Future research should explore the utility and overall impact of such framework in allied health service delivery.

## Background

The sustainability of Australia’s current health system and level of service it provides is an increasing concern for federal and state governments. Multiple factors are involved, such as increasing availability of, and demand for, advanced technology services, an ageing population (more older people surviving, but with the chronic and multi-morbid diseases of ageing impacting on their independence, and quality of life), rapid advances in technology, and the ongoing issues meeting supply of and demand for healthcare providers. It is projected that under the current system, health care expenditure will increase to between 12 and 15% of the gross domestic product over the next 30 years [1].

Generally underpinning the health care system are pressures to reduce costs, increase access and affordability of services, and provide greater accountability. There is also an increasing recognition worldwide of the need to examine how healthcare providers practice, and justify their performance and productivity. Measuring and monitoring aspects of the services provided by healthcare practitioners has therefore become a major concern not just in Australia but in many other countries [2]. Performance evaluation *‘seeks to monitor, evaluate and communicate the extent to which various aspects of the health system meet their key objectives’* [3]*.* Performance is therefore an important indicator of how well a healthcare system is progressing towards its goals, and helps identify strengths and weaknesses to improve future performance [4]. There is international evidence to suggest that organisations which do not integrate ongoing performance evaluation into their system tend to experience lower than expected performance improvements, as well as higher dissatisfaction and turnover of staff [5].

Allied health services have been increasingly highlighted over the last five years as essential primary, sub-acute and tertiary services which could contribute significantly more to Australia’s healthcare system than they are currently doing [6]. Allied health is an umbrella term used to describe a range of health disciplines, other than medicine and nursing, which provide therapy, organisational and scientific services [6]. Commonly included under this umbrella are disciplines such as physiotherapy, occupational therapy, podiatry, speech pathology, social work, dietetics and nutrition, psychology, audiology and psychology. Although there have been attempts to define allied health [7,8], there remains a lack of an internationally recognised definition because of the range and complexity of services delivered by the disciplines listed under the allied health umbrella. The lack of definitive definition for allied health precludes a comprehensive understanding of allied health quality service issues [9].

The design of an effective performance evaluation strategy is fundamental to aligning allied health organisation’s operations with its strategic direction. It involves an ongoing cyclical process of information gathering, analysis and action at different levels—the workforce, consumers of care, and organisation in which the services are provided. However, there is no agreed approach to measuring or monitoring allied health service performance. This appears to be, in part, due to the *diversity of disciplines which fall under the allied health umbrella, the variability in roles and tasks these disciplines undertake, and lack of standard data items, data collection processes, and dedicated support systems to capture the range of services that allied health provides* [6]. Therefore, it is clear that there is no ‘one size’ fits all approach that can be used to measure allied health service performance. It also highlights the potential challenges and barriers associated with performance evaluation of allied health practitioners, specifically in terms of selection of performance measures, data collection, and implementation of an effective performance evaluation strategy.

The aim of this review was to examine the literature on performance evaluation in healthcare to assist in the establishment of a framework that can guide the measurement and evaluation of allied health clinical service performance. This review determined the core elements of a performance evaluation system, tools for evaluating performance, and barriers and challenges to the implementation of performance evaluation.

## Methods

The following section describes the search strategy used in this review.

### Research design

A systematic review of the literature using a narrative synthesis approach was undertaken.

### Criteria for considering studies in the review

All peer-reviewed publication types including literature reviews, quantitative studies (e.g. evaluation studies, observational studies), qualitative studies, mixed-methods studies and discussion papers were included in the review. From an initial scoping of the literature, it was found that very few articles specific to allied health performance evaluation were published. As this will provide very limited evidence base, publications which focused on any allied health performance evaluation or those which examined performance in health care in general were considered in the review. However, studies which focused on nurses or physicians only were excluded, as were those studies which described assessment of student-related performance or those which focused on improving educational curriculum. Publications that described performance evaluation for individual practitioners, organisations or at a national level were reviewed. Table 1 provides a summary of the inclusion and exclusion criteria.

**Table 1:** **Criteria for inclusion of studies in the review**

**Inclusion criteria**	**Exclusion criteria**
• Articles which described performance evaluation in any	• Articles which focused only on nurses or doctors
• allied health discipline or health in general, at any level (e.g. individual, departmental)	• Commentaries, conference abstracts and non-peer reviewed literature
• Literature reviews, primary studies (e.g. quantitative, qualitative), and discussion papers	
• English articles	
• Articles published between 2000-2013	

### Search strategy

Five electronic databases were used to search for relevant articles: MEDLINE, Embase, CINAHL, PsychInfo, and Academic Search Premier. The following search terms were used for MEDLINE and adapted for the other databases: *performance measurement, performance evaluation, performance assessment, performance monitoring, performance appraisal* and *allied health, healthcare, health* care, including subheadings. Table 2 shows an example of the search strategy in one of the databases. Reference lists of included articles were searched for relevant references not found in the electronic database search.

**Table 2:** **Example of a search strategy**

**Database**	**Search #**	**Search term**	**Hits**
**OVID:** Medline *Keyword search*	1.	“performance measurement”.mp. or performance measurement system/	962
	2.	“performance evaluation”.mp.	2992
	3.	“performance assessment”.mp.	1387
	4.	“performance monitoring”.mp.	717
	5.	“performance appraisal”.mp.	4449
	6.	OR/1-5	10214
	7.	“allied health”.mp.	13874
	8.	(“healthcare” or “health care”).mp. [mp = title, abstract, original title, name of substance word, subject heading word, keyword heading word, protocol supplementary concept word, rare disease supplementary concept word, unique identifier]	654805
	9.	OR/7-8	664947
	10.	6 AND 9	2106
	11.	Limit 10 to English and yr = “2000 - 2013”	1144
	12.	Minus duplicates	698

Limits were used to include only articles written in the English language and published between 2000–2013. The inclusion of articles within this period aimed to capture articles which were published following the emergence of a seminal paper [10] which set the standards for health care quality.

### Selection of studies

The titles and abstracts of studies identified by the search strategy were independently assessed for eligibility by two reviewers (LL, KG). Full text copy of the different studies considered to be potentially relevant for the review was then retrieved for further examination. The same reviewers (LL, KG) independently examined the studies against the selection criteria for inclusion in the review. Disagreements about the inclusion or exclusion of particular studies were resolved by discussion between the two reviewers and confirmed with a third reviewer (SK).

### Quality assessment

The study design of included publications was determined using the National Health and Medical Research Council (NHMRC) evidence hierarchy [11]. The methodological quality of individual studies was not assessed as this review aimed to examine the evidence regarding the core elements of, tools for, and barriers to performance evaluation rather than a review of effectiveness of performance evaluation systems. In addition, the majority of publications included in the review were discussion papers, which did not allow the use of a structured critical appraisal tool.

### Data extraction and analysis

Content analysis was used in this review to provide a framework for data extraction and guide the synthesis of data from individual studies. This approach was chosen as it can be used to synthesise data from a diverse range of literature [12] and is considered to be an appropriate method when data available are descriptive in nature [13], which was the case in this systematic review. Content analysis is an established method in research and involves developing categories ‘a priori’ and coding the individual studies against these categories [14,15].

The content analysis approach for this review drew on the systematic review techniques reported by Evans & Fitzgerald [14]. A series of steps were used to gather and analyse evidence from individual studies. Firstly, all included articles were read and re-read to develop an initial impression of the body of literature. Two reviewers worked collaboratively to identify recurring ‘key issues’ form individual studies, creating a list of initial categories which informed the development of an extraction tool. Using a random sample of included studies, the initial categories were tested, revised as necessary, and then finalised by the two reviewers (LL and SK). The first author (LL) coded the individual studies and extracted information based on the final list of categories, which were then double-checked by another reviewer (SK) for accuracy. Individual studies each contributed data related to a number of categories relevant to performance evaluation.

Four major categories and six sub-categories were used:

1. Purpose of performance evaluation.
2. Core elements of performance evaluation.
  Prioritising clinical areas for evaluation.
  Setting the goals for performance evaluation.
  Selecting performance measures.
  Identifying types and sources of information.
  Undertaking performance evaluation.
  Reporting of results.
3. Tools for evaluating performance.
4. Barriers to implementation of performance evaluation

As the majority of the literature found for this review involves healthcare in general rather than allied health-specific, a theoretical framework describing allied health service delivery was used to contextualise the findings to allied health [16]. This framework describes allied health services in terms of *‘what allied health does’* (considers allied health roles, responsibilities and tasks)*, ‘how allied health does it’* (time lines, performance and organization of allied health care) and *‘what happens’* (short term and long term outcomes from allied health services) [16].

## Results

### Literature base

**Figure 1:**
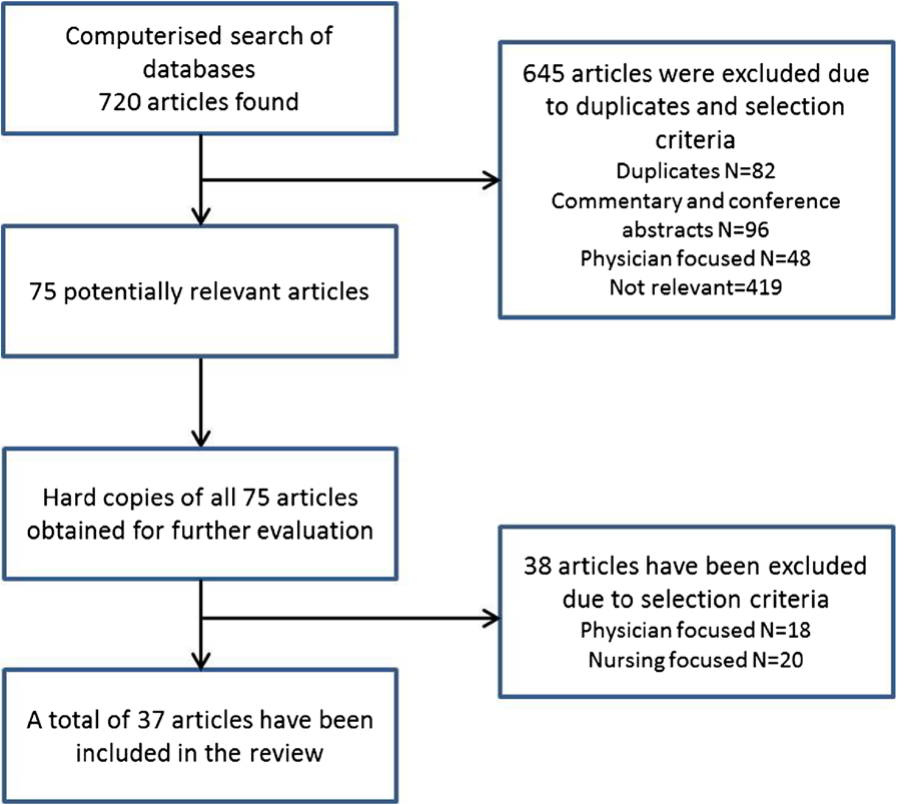
**Consort diagram of selection procedure.**

The database search yielded 720 articles, of which 645 were excluded due to duplicates and selection criteria. Full text copies were retrieved for the remaining 75 articles for further comparison against selection criteria. After scrutiny, 38 were further excluded; leaving 37 articles for inclusion in the review. A consort diagram for the literature search is shown in Figure 1.

Of the 37 articles that were reviewed, only five were specific to allied health, with the remaining publications reporting on health care in general. The information presented in most articles is based on USA (United States of America) and UK (United Kingdom) experience, with only a few describing performance measurements in the Australian context, Asia and other European countries.

### Hierarchy of evidence

The included studies comprised discussion papers (46%), literature reviews (16%), mixed-methods studies (13%), case studies (11%), observational studies (8%), audits (3%), and qualitative study (3%).

### Evidence map

Table 3 shows the coding assigned to each of the individual articles included in the systematic review, summarising the evidence sources for each of the identified categories.

**Table 3:** **Evidence map**

**Evidence source**	**Purpose of performance evaluation**	**Core components of performance evaluation**						**Tools**	**Barriers**
		**Prioritise clinical area**	**Set goals**	**Select performance measures**	**Identify type & source of information**	**Undertake performance evaluation**	**Report results**		
Arnold & Pulich [17]	√			√		√		√	√
Bannigan [18]				√			√	√	
Bente [19]				√					
Beyan & Baykal [2]			√	√	√				
Chandra & Frank [20]	√			√		√		√	√
Colton [21]							√		
Derose & Petiti [22]	√			√	√				
Doherty & DeWeaver [23]	√					√		√	
Geddes & Gill [24]	√			√				√	√
Geraedts et al. [25]		√		√	√	√			
Gregory 2000 [26]	√								
Hamilton et al. [27]				√	√			√	√
Harp [28]								√	
Johansen et al. [29]				√				√	
Jolley [30]	√			√	√				
Kilbourne et al. [31]				√	√				
Koch et al. [32]								√	
Kollberg et al. [33]	√								√
Koss et al. [34]	√								
Loeb [35]			√	√					√
Mainz [36]	√			√					
Mainz [37]		√							
Manderscheid [38]	√								
Mannion & Goddard [39]	√		√	√					
Mant [40]	√								
Marshall & Davies [41]		√		√					
Nuti et al. [42]	√		√		√		√		
Perrin [43]				√					
Purbey et al. [4]				√	√				
Roper & Mays [44]				√					
Salvatori et al. [45]							√		√
Sibthorpe & Gardner [46]			√	√					
Sund et al. [47]	√								
Tawfik-Shukor et al. [48]	√		√	√					
Van der Geer et al. [49]				√					
Vasset et al. [50]				√					
Veillard et al. [51]			√	√					

### Purpose of performance evaluation

Sixteen articles explicitly reported about the purpose of performance evaluation in health care [17,20,22-24,26,30,33,34,36,38-40,42,47,48]. The literature reports numerous reasons for undertaking performance evaluation and for the majority of the reviewed studies performance evaluation denotes measurement of health care quality. Obtaining an accurate insight about the quality of care and promoting improvement in terms of health service delivery [22,30,36,38-40,42,47,48], administration and operational and financial management have been identified as one of the key roles of performance evaluation [30]. The ultimate goal for which is to improve health outcomes by stimulating improvements in health care. The premise of performance evaluation is to assist health practitioners or organisations identify issues that require attention or opportunities for improvement, and recognise satisfactory performance and effective practices. Once they are identified, strategies can be taken to foster improvement and achieve the desired outcomes.

**Figure 2:**
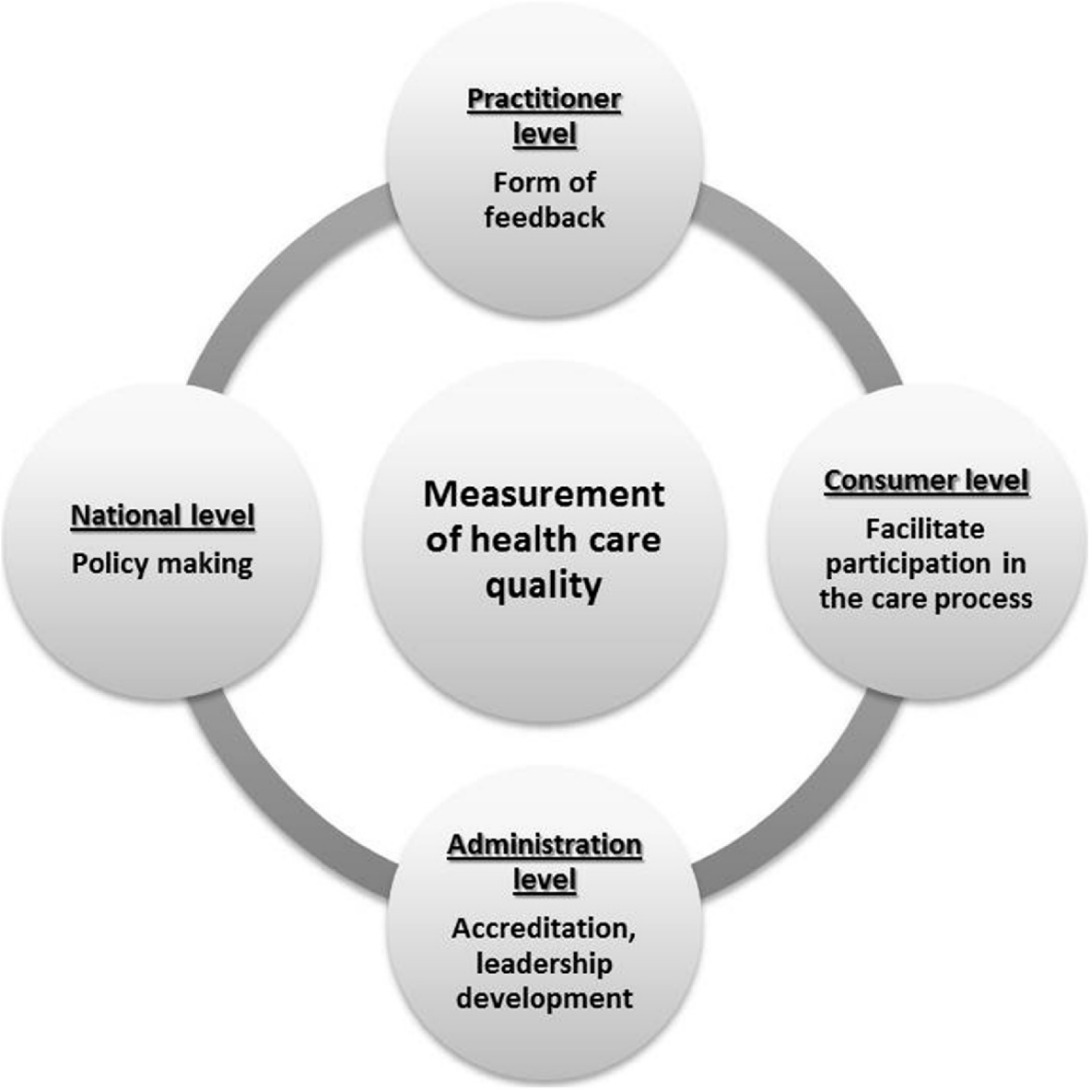
**Key domains of performance evaluation.**

In addition to improving the quality of health care, there are many other reasons for undertaking performance evaluation and they can be categorised based on the perspectives of different stakeholders. From a practitioner perspective, performance evaluation can be an effective tool in providing objective feedback in order to validate their skills and practice or facilitate corrective action if poor skills are demonstrated, or as a medium to correct or reward performance [17,20,23,24,26,34,40]. It can also assist in identifying professional development needs and in fulfilling professional regulatory body obligations [24]. At a consumer level, performance evaluation provides clients with information that can facilitate choice of health care provider [36,40] and allows them to participate in the improvement of care delivered to them [23]. For senior personnel, managers or administrators, performance evaluation can assist in meeting accreditation standards and third-party contractual standards [24,36]. It can also facilitate leadership development, and inform human resources decisions (e.g. pay increases, promotions) [17,20,24]. Kollberg et al. [33] defines performance evaluation as ‘*the process of collecting, computing, and presenting quantified constructs for the managerial purposes of following up, monitoring, and improving organizational performance* [33]*.’* Finally, at the national level, performance evaluation data can inform policy making and assist with formulating strategies at a regional or national level [40]. Figure 2 summarises the different functions of performance evaluation.

### Core elements of a performance evaluation system

**Figure 3:**
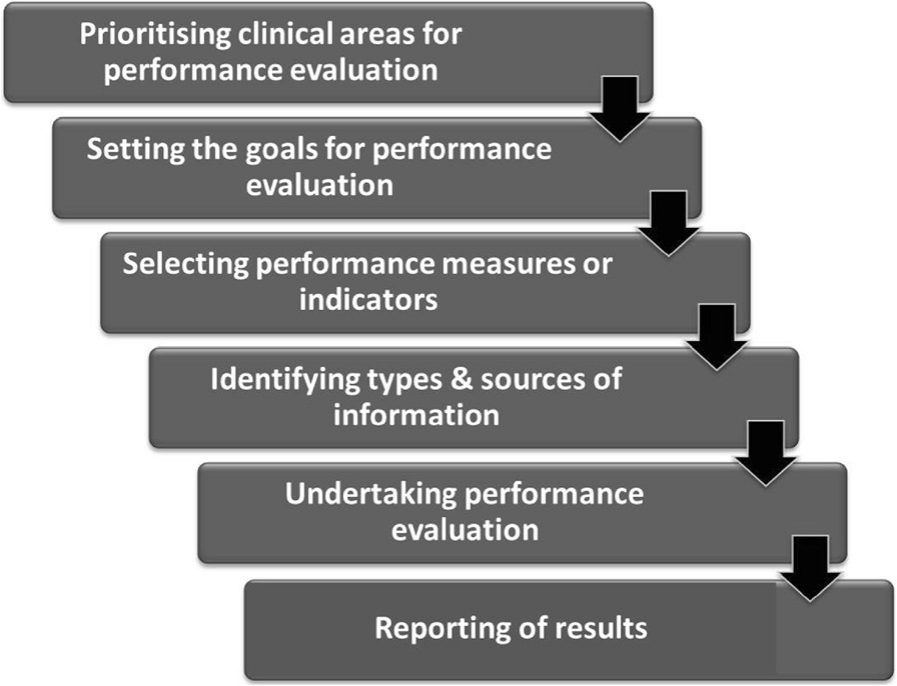
**Core elements of performance evaluation.**

A critical examination of the literature identified key steps (as shown in Figure 3) to a successful performance evaluation system. Twenty eight articles [2,4,17-25,27,29-31,35-37,39,41-46,48-51] described these key steps which are summarised in the following section.

#### Prioritising clinical areas for performance evaluation

Undertaking performance evaluation can be a laborious and time-consuming process, and for it to be meaningful, carefully selecting a clinical area for evaluation is very important. The literature proposed several criteria for selecting aspects of care suitable for performance evaluation, and these include areas which:

- Are important and relevant to the group for which the performance evaluation system is being produced (as healthcare ‘quality’ is viewed in various ways by different groups of stakeholders) [25,41].
- Are problem-prone and with high frequency of occurrence, or those suspected of overuse, underuse, or misuse [25,37,41].
- Have strong financial impact [25,37,41].
- Have the potential to improve health care delivery and outcomes [25,37].
- Have recently undergone major changes [25].
- Have proven and significant variation in quality of service among health care providers, or where there is evidence that the quality of service is suboptimal [25,37,41].
- Are considered high risk for patients [25].

#### Setting the goals for performance evaluation

The basic principle of good performance evaluation is the upfront development of strategic measurement goals [35,39,46]. The goal of evaluation is typically targeted to improve the following domains: acceptability, accessibility, appropriateness, care environment and amenities, continuity, competence or capability, effectiveness, improving health or clinical focus, expenditure or cost, efficiency, equity, governance, patient-centeredness, safety, sustainability, timeliness, and utilization [2,42,48,51]*.* A performance evaluation activity usually targets more than one dimension, and is generally designed to address the needs of the stakeholders.

#### Selecting performance measures

A performance measure or indicator, also known as quality indicator, refers to ‘*a quantitative measure that can be used to monitor and evaluate the quality of important governance, management, clinical and support functions that affect patient outcomes* [36].’ It measures the extent to which set goals or targets are achieved.

**Figure 4:**
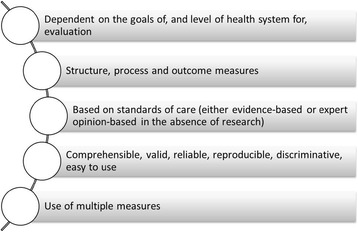
**Factors to consider when selecting performance measures.**

Figure 4 summarises the basic factors to consider when selecting performance measures.

- The performance measure should correspond to one or more of the target dimensions or goals (i.e. acceptability, accessibility, appropriateness etc.) and is determined based on the level of health system being evaluated. At the practice or individual practitioner level, the performance measures can be developed from goals and objectives, which should be in line with the individual’s work duties, and the strategic and operational goals of the organisation [17,20,24,30]. Individual practitioners’ goals should be jointly established by the manager and the practitioner, as this will provide the opportunity for the manager to engage in interim planning with the practitioner [17]. At the organisational level, performance measures should be linked to the strategic planning of the service and the overall organisation values and standards [4,20,30]. At the national level, performance measures should capture outcomes which are broad in scope [30].
- Performance measures are related to structure, process and outcomes [52] and these quality concepts have been reported in performance evaluation studies [2,22,29,31,36,39,43,44,46,48,49]. The ‘structure’ measures evaluate the means and resources used by the health system to deliver services [2,36]. Quantity and quality of health personnel (staffing), physical facilities, equipment and infrastructure, and existence of regulatory programs are considered as structural measures [46]. ‘Process’ measures examine the interaction between service providers and consumers [2]. It is concerned with activities which are carried out in relation to diagnosis, treatment, rehabilitation and care. Process measures assess what the health practitioner did for the patient and how well it was done [46]. ‘Outcome’ measures examine the change in patients’ health status, which can be attributed to the effectiveness of the treatment. It is comprised of both physical and perceived benefits such as improvement in health status, satisfaction from the health service, receiving health related information and changing habits in maintaining personal health [2,46].
- Performance measures are based on standards of care, which can be evidence-based or, in the absence of scientific evidence, determined by an expert panel of health practitioners [19,27,36].
- Performance measures must be clear, valid, reliable, reproducible, discriminative and easy to use [4,18,22,25,36,41,44,50,51]. They should be comprehensive yet practically relevant and meaningful [24,36].
- The use of multiple measures is favored over a single measure in order to obtain a comprehensive picture of health care [35]. Single measures are, in most cases, limited in scope thereby reducing their utility to relevant stakeholders.

#### Identifying types and sources of information

Performance evaluation should obtain information or data from several perspectives, (i.e. multi-feedback) as this will provide a richer assessment of performance compared to a single source [4,27,30,31,42]. This should involve representatives from specific stakeholder groups depending on the level of health system being evaluated [25,30].

Information can be obtained from various sources, such as information systems, reports, surveys and records. Data types are usually categorised as clinical data, administrative data and patient-based data [2,22]. Clinical data includes information which can be obtained from all types of medical records or medically oriented sources such as outcome measurements reported in patient charts, discharge reports, diagnostic reports. Administrative data are related to health costs including billing and claims. Patient-based data refer to the information collected directly from patients through questionnaires or interviews.

#### Undertaking performance evaluation

When undertaking performance evaluation, the objectives, procedures, participants (target groups), materials (e.g. training materials, interpretation guides, etc.), and premises for performance evaluation should be clearly identified and documented [25]. A schedule for performance evaluation that works well with the practice is recommended [23]. Evaluator training is also a key factor to conducting effective performance evaluation [17,20]. Training has been reported to improve consistency and develop confidence with the use of evaluation instrument [20]. All ‘evaluators’ or anyone completing the measurement must be instructed about the performance measurement process [17].

#### Reporting of results

Reporting of results should be built into the performance evaluation system [18,42]. The results serve as feedback to health practitioners and their organisation, either as recognition for good performance or as a prompt for further improvement or development, which can increase service performance or work motivation [21]. While much of the data collected in health care organisations can stand alone in providing useful information, additional information can be obtained when comparative data is also presented. When available, norms, standards and benchmarks provide opportunities to compare data to external sources [45].

### Tools for evaluating performance

**Figure 5:**
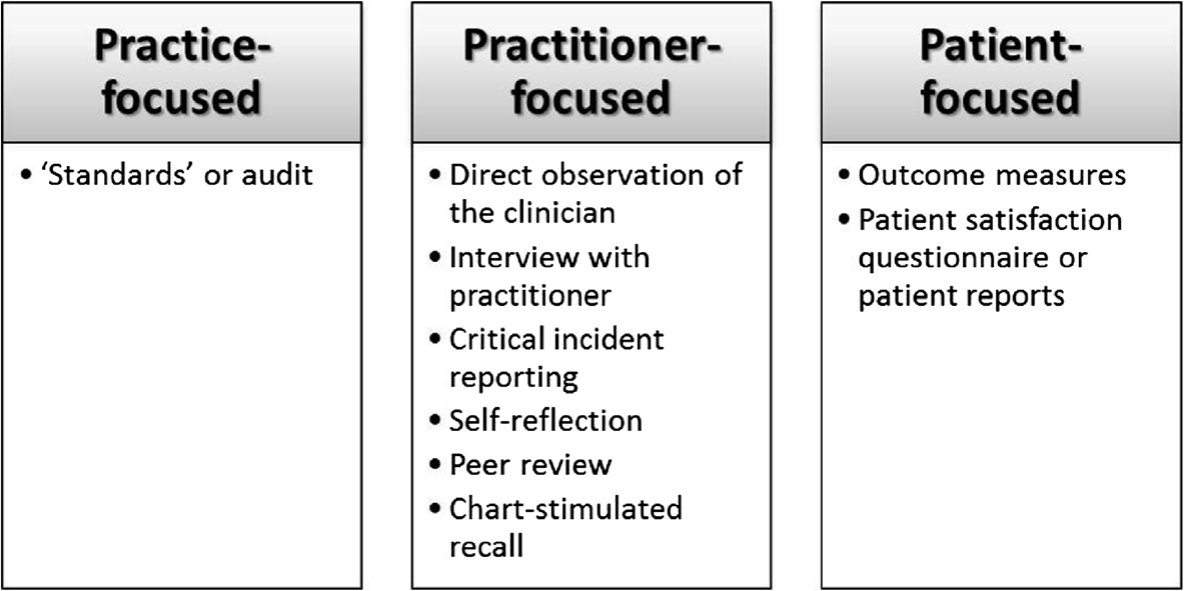
**Tools for measuring performance.**

Nine articles [17,20,23,24,27-29,32,49] reported a wide range of tools for evaluation, often comprising the use of more than one instrument. The choice of tools is dependent on several factors including but not limited to the level of health system for evaluation, objectives of evaluation and target participants. Figure 5 illustrates the different performance evaluation tools described in the literature.

‘Standards’ or audit is a practice-focused tool in which the performance data collected are compared with pre-determined standards of practice, locally or nationally [18,27]. These standards define the level of performance required for the successful achievement of work expectations and specify what the consumers can expect from the practitioners [27].

Practitioner-focused tools consisted of the following: direct clinical observation of the clinician in the patient’s setting [20,23,24], interview with practitioner [24], critical incident reporting [18], self-reflection or self-appraisal [24], peer review or appraisal [17,18,20], and chart-stimulated recall [28].

Patient-focused tools include the use of outcome measures to collect information about patient health status [29,53]. Outcome measures are used to determine change in patients’ status over time. They provide clinicians with feedback on patient outcomes, allow progress of status to be effectively communicated to patients, and promote treatment planning [32]. Routine outcome measurement can also support or justify the interventions administered to patients, and provide supporting evidence to funding bodies [32]. In addition to the use of outcome measures is the use of patient/client satisfaction questionnaire [18], or patient reports (verbal reporting to the practice team [23], or actual complaints from clients [18]. Koch et al. [32] suggest that patient data may be used to demonstrate accountability, feedback to individual practitioner, staff supervision, meet accreditation requirements, enhance staff morale, and support budget requests [32].

### Barriers to implementation of performance evaluation

While there are significant benefits associated with performance evaluation, the literature also highlighted barriers and challenges to its implementation. Findings from seven articles [17,20,24,27,33,35,45] which described these barriers and challenges were summarised. The time required [24] and cost associated [20,27,35] with the process of undertaking performance evaluation were reported as significant barriers. The time and manpower needed to support performance evaluation may be constrained by a health care financial system that places limitations on reimbursements [45]. Personality conflict between managers/supervisors and individual practitioner was also identified as a major impediment for performance evaluation [17,24]. There may also be resistance from practitioners who are skeptical about the validity and usefulness of performance evaluation data. Difficulty in motivating personnel and heads/managers of health departments was also raised as an important barrier to performance evaluation [33].

### Contextualising performance evaluation in allied health

**Figure 6:**
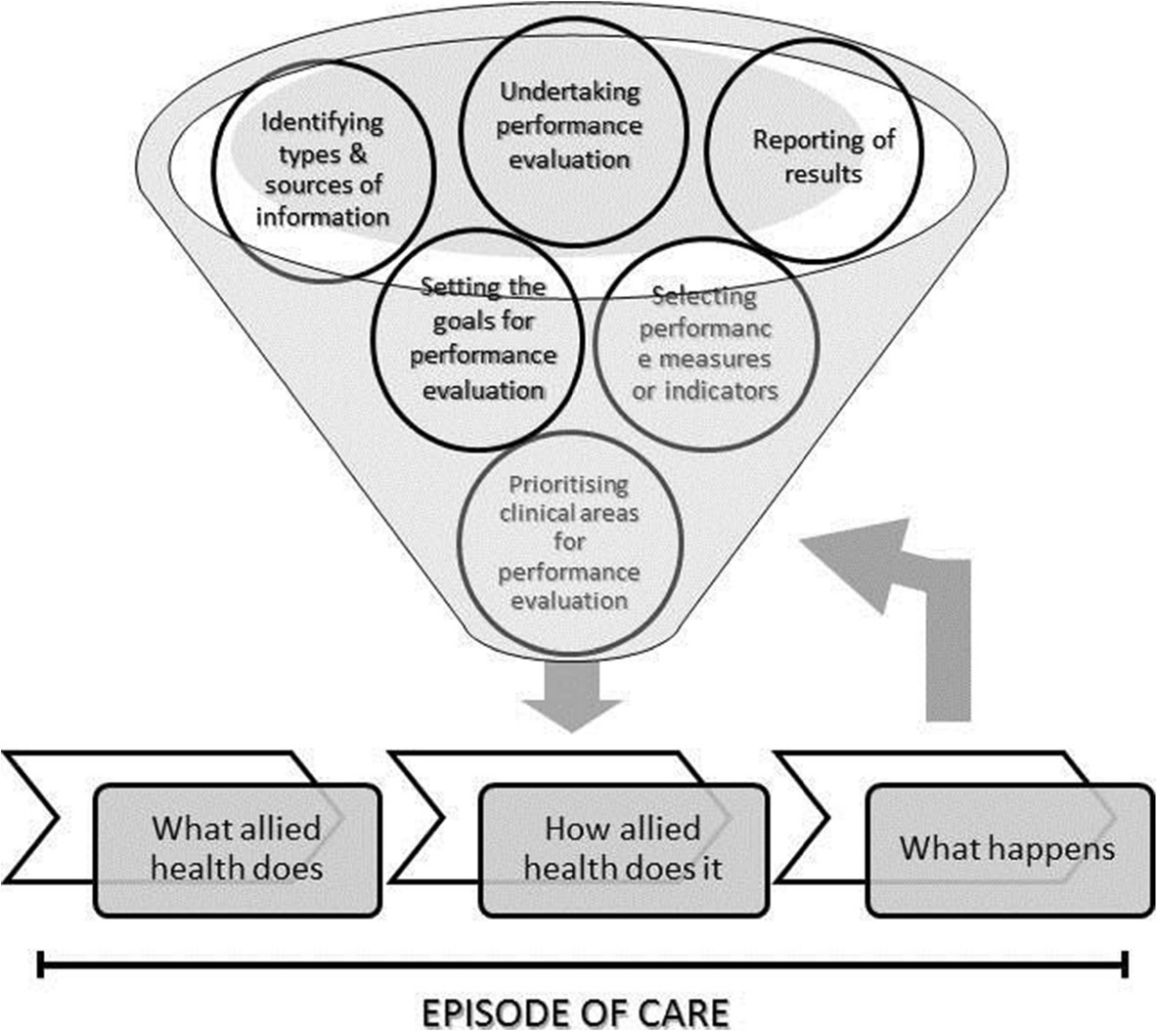
**Core elements of performance evaluation aligned with allied health service.**

The evidence base found in this review appears to be generalizable to healthcare and not necessarily specific to allied health. However, by using the framework proposed by Grimmer et al. [16], which described the complex, episode of care nature of allied health services [16], a model of performance evaluation strategy for allied health is proposed (see Figure 6). In allied health, service delivery is described in terms of episodes of care, which is defined as ‘*comprising all those occasions of service provided to the one patient for the one condition in the one allied health service, using the one referral* [16].’ The core elements of performance evaluation were mapped against the framework for allied health service. In this model, performance evaluation feeds into ‘how allied health does it’, which in turn influences ‘what happens’ in an episode of care. The performance of the discipline/s (i.e. ‘how allied health does it’) and how care is delivered to clients determines the outcomes of allied health services.

## Discussion

Performance evaluation is an integral part of health care. Its primary aim is to measure the quality of health services with the ultimate goal of improving health outcomes. Measurement and evaluation of allied health performance and quality of services is in its infancy and the complexity of the services they provide contributes to the challenges associated with the process. In allied health, the individual disciplines have different purposes, ways of operating, stakeholders, outcomes and quality measures. As such, there is no one-size-fits-all approach for performance evaluation that can be recommended to all allied health care settings. However, the literature suggests that there are core elements involved in performance evaluation which include prioritising clinical areas for measurement, setting goals, selecting performance measures, identifying sources of feedback, undertaking performance measurement, and reporting the results to relevant stakeholders. The literature describes performance evaluation as multi-dimensional, requiring information or data from more than one perspective to provide a rich assessment of performance. A range of tools or instruments is available to capture various perspectives and gather a comprehensive picture of health care quality. This review, while it is primarily targeted to allied health services, generates findings that are broad and appear to be generalisable to a wide range of healthcare settings (e.g. medical, nursing, allied health), both locally and internationally. The premise that performance evaluation should be context dependent and designed to meet the unique requirements of the health care system holds true and remains valid, regardless of the healthcare setting being evaluated. Therefore, the findings of this review are not necessarily unique to allied health but may also be used to inform performance evaluation in the wider health system.

Performance evaluation is reported for different levels of the health care system, ranging from individual practitioners to geographical (e.g. regional or state) locations. Performance evaluation of an individual practitioner can promote ownership of the quality of care provided to clients [43]. However, differences in performance between practitioners are often the result of *‘random fluctuations rather than real differences in quality of care* [41].*’* On the other hand, by examining a state level performance, statistical problems, which are common in individual level assessment, are not an issue. At this higher level, however, practitioners do not feel a sense of ownership for their performance and are therefore not likely to be motivated to change or improve their practice [41]. Marshall and Davies [41] suggest a mid-level assessment, which involves small functional groups of health professionals, ranging from individual hospitals to groups of practices [41]. In allied health, for example, an evaluation of the performance of podiatrists in foot screening for diabetic patients in a specific hospital is more ideal than evaluation of podiatry practice across a range of clinical conditions.

The selection of clinical area for evaluation is often dependent on what data or information is readily available, and as such the evaluation process starts by determining what data are accessible within the practice or organization [35,41]. This is then followed by the identification of goals, which align with the available performance data—an approach that seeks to minimise the collection of further data. While this would be cost-effective, it violates the basic principle of having to establish goals prior to the development of the evaluation system or process. If performance evaluation is to be meaningful, the clinical area for performance evaluation should be carefully selected and be based on explicit criteria [25,36]. Loeb [35] claims that a more rational and appropriate approach would be to define the evaluation goals relevant to the clinical area, and then determine whether reliable data exist to support such goals [35]. Additional data collection can then be applied when required and if it outweighs the time and costs associated with the process [35]. It is always important to obtain the commitment of the chief executive and management team, as this is critical to the successful implementation of performance evaluation systems [45]. The management plays a key role in performance evaluation as they will articulate the system or organisation’s vision of quality, ensure that there is an infrastructure and systematic approach, and make resources available to support the process [45].

The identification and selection of appropriate performance measures (i.e. whether to use outcome or process measures, or both) is one of the most challenging activities for those who undertake performance evaluation. Mant [40] argued that in instances where health services have a major impact on outcome, use of outcome measures as performance indicators is appropriate, provided that the data collected can be interpreted reliably [40]. Conversely, in situations where factors such as lifestyle, co-morbidities, socio-economic circumstances rather than health care play a major role in health outcomes, process measures are preferred [19,40]. This does not mean, however, that outcome data should not be collected, just that it should be collected within context. In other words, what seems to be the best solution is to combine process and outcome measures which are tailored to local circumstances and priorities [39]. The identification of specific outcome and process indicators are then based on standards of care, or in the absence of scientifically-based evidence, determined by an expert panel. Performance evaluation typically involves a combination of measures, which are comprehensible, clear, valid, reliable, reproducible, discriminative and easy to use. Grimmer et al. [16] proposed quality measures relevant to allied health therapy services which capture the elements of ‘what allied health does’, ‘how allied health does it’, and ‘what happens [16]’.

The data or information required to measure process and outcomes could be retrieved from various sources including clinical or medical records, administrative data and patient reports. There is also a range of tools available that can be used to monitor performance [17,20,23,24,27-29,53]. The analysis of data and reporting of results should then lead to the recognition of good performance, improvement of poor performance and modification of the performance evaluation system if required. There is no point in conducting a performance evaluation if the results are not followed through. There should be a clear plan of action that needs to be agreed upon by relevant stakeholders in order for performance evaluation to be meaningful and worthwhile. Improvement to the performance evaluation system and attention to barriers and challenges can then facilitate its effective and sustainable uptake by allied health care practitioners and organisations.

As with any other studies, this review has a number of limitations which should be considered when interpreting the results. First, there are limitations to the search and it is possible that articles could have been missed as the search strategy did not include terms which referred to ‘quality of healthcare’ which was considered in the literature as an important component of performance evaluation. Second, the key concepts or domains identified in this review may have been influenced by the perspectives of the reviewers and it is possible that if a different perspective was obtained, a different set of concepts or domains would have been found; a validation study may be required to confirm the findings. Despite these limitations, the findings presented in this review provide valuable insights to clinicians, managers and health service researchers that can assist with the development of a broad framework for undertaking performance evaluation.

## Conclusions

Every allied health care delivery system is unique, and has different performance needs and will therefore require different approaches. This paper provides a synthesis of the published literature regarding the key elements of conducting performance evaluation which can be applied in an allied health care setting. Underpinning an effective performance evaluation system are core processes that include prioritisation of clinical area for evaluation, upfront articulation of goals, careful identification of performance measures, mapping of measures to information sources, and analysis of performance data and reporting of results. A mid-level assessment that involves small functional groups of health professionals or practices is recommended as it promotes a sense of ownership of professional performance and effective team work. A careful examination of barriers to performance evaluation and subsequent tailoring of strategies to overcome these barriers should be undertaken to achieve the aims of performance evaluation. The findings of this review should inform the development of a standardised framework that can be used to measure and evaluate allied health performance. Future research should explore the utility and overall impact of such framework in allied health service delivery.
